# Retraction Note to: Differential regulation of wild-type and mutant alpha-synuclein binding to synaptic membranes by cytosolic factors

**DOI:** 10.1186/s12868-021-00644-1

**Published:** 2021-06-03

**Authors:** Sabine Wislet-Gendebien, Naomi P. Visanji, Shawn N. Whitehead, Diana Marsilio, Weimin Hou, Daniel Figeys, Paul E. Fraser, Steffany A. L. Bennett, Anurag Tandon

**Affiliations:** 1 Centre for Research in Neurodegenerative Diseases, University of Toronto, Toronto, ON M5S 3H2 Canada; 2 Centre for Cellular and Molecular Neurobiology, University of Liege, 4000 Liege, Belgium; 3 Neural Regeneration Laboratory, Ottawa Institute of Systems Biology, Department of Biochemistry, Microbiology, and Immunology, University of Ottawa, Ottawa, K1H 8M5 Canada; 4 Institute for Biological Sciences, National Research Council of Canada, Ottawa, K1A 0R6 Canada

## Retraction to: BMC Neurosci (2008) 9:92 10.1186/1471-2202-9-92

The Editor has retracted this article. Concerns have been raised with a number of figures, specifically:

- Figure 1C: The right three blots of the upper left hand band appear to be identical to the right three blots of the upper right hand band.
- Figure 5D: the Synaptophysin panels for both 1 minute-cross linking and 3 minute cross-linking appear to be identical.
- Figure 5D: the a-synuclein panels appear to look very similar.

The Editor therefore no longer has confidence in the reliability of the data reported in the article.

Sabine Wislet-Gendebien does not agree to this retraction. Naomi P Visanji, Shawn N Whitehead, Diana Marsilio, Weimin Hou, Daniel Figeys, Paul E Fraser, Steffany AL Bennett and Anurag Tandon agree to this retraction.

